# Hemodynamic Significance of Middle Cerebral Artery Stenosis Associated With the Severity of Ipsilateral White Matter Changes

**DOI:** 10.3389/fneur.2020.00214

**Published:** 2020-04-15

**Authors:** Hui Fang, Xinyi Leng, Yuehua Pu, Xinying Zou, Yuesong Pan, Bo Song, Yannie O. Y. Soo, Thomas W. H. Leung, Chunxue Wang, Xingquan Zhao, Yilong Wang, Yongjun Wang, Ka Sing Wong, Liping Liu, Yuming Xu, The CICAS Study Group

**Affiliations:** ^1^Department of Neurology, The First Affiliated Hospital of Zhengzhou University, Zhengzhou, China; ^2^Department of Medicine and Therapeutics, Prince of Wales Hospital, Shatin, Hong Kong; ^3^Department of Neurology, Beijing Tiantan Hospital, Capital Medical University, Beijing, China; ^4^China National Clinical Research Center for Neurological Diseases, Beijing, China; ^5^Center of Stroke, Beijing Institute for Brain Disorders, Beijing, China; ^6^Beijing Key Laboratory of Translational Medicine for Cerebrovascular Disease, Beijing, China

**Keywords:** ischemic stroke, intracranial atherosclerosis, magnetic resonance angiography, imaging, white matter changes

## Abstract

**Background:** Previous studies conflicted in the association between intracranial atherosclerotic stenosis (ICAS) and the severity of white matter changes (WMC).

**Aims:** We aimed to investigate the relationships between the severity of luminal stenosis and the hemodynamic significance of middle cerebral artery (MCA) stenosis, and the severity of ipsilateral WMC.

**Methods:** In this cross-sectional study, patients with a recent ischemic stroke or transient ischemic attack and a 50–99% MCA-M1 stenosis in the Chinese Intracranial Atherosclerosis study cohort were analyzed. The post- to pre-stenotic signal intensity ratio (SIR) was obtained in time-of-flight MR angiography (MRA) to represent the hemodynamic significance of MCA-M1 stenosis, with a lower SIR indicating a hemodynamically more severe lesion. The severity of ipsilesional WMC was assessed by an age-related WMC (ARWMC) scale in T2-weighted fluid attenuated inversion recovery MR imaging. The relationships between the degree of MCA-M1 stenosis, SIR, and ipsilesional ARWMC scale were analyzed. The MCA-M1 lesion with a higher percentage of stenosis was chosen for analyses in patients with bilateral MCA-M1 stenoses.

**Results:** Among 180 subjects (mean age, 64 years), a lower SIR of MCA-M1 stenosis (Spearman correlation coefficient, −0.543; *p* < 0.001), but not the degree of stenosis (*p* = 0.93), was significantly linearly correlated with a higher ipsilateral ARWMC. Multivariate ordinal logistic regression identified older age (OR = 1.037; 95% CI, 1.008–1.066; *p* = 0.011) and lower SIR (OR = 0.010; 95% CI, 0.002–0.058; *p* < 0.001) as independent predictors for more severe ipsilateral WMC.

**Conclusion:** Patients with hemodynamically more severe ICAS are more likely to have more severe ipsilateral WMC. Longitudinal studies with sequential imaging exams may further reveal the impact of hemodynamic significance of ICAS on the development and progression of WMC.

## Introduction

White matter changes (WMC) have been linked to cognitive dysfunction, gait impairment and falls, depression, and increased risk of future stroke (1–4), but the pathophysiology underlying development and progression of WMC are not fully elucidated. Previous studies showed conflicting results regarding whether extra- or intra-cranial atherosclerotic disease is associated with WMC, and which is more closely correlated with WMC (5–8).

We had previously proposed an index termed signal intensity ratio (SIR) to quantify the hemodynamic significance of intracranial atherosclerotic stenosis (ICAS), defined as the ratio of distal (post-stenotic) and proximal (pre-stenotic) signal intensities (SIs) obtained in the vessel lumen in time-of-flight magnetic resonance angiography (MRA), which has been recently validated against CT perfusion measures (9–12). In the present study, we aimed to investigate the associations of severity of luminal narrowing and hemodynamic significance of middle cerebral artery (MCA) stenosis by SIR, with the severity of ipsilateral WMC, in ischemic stroke or transient ischemic attack (TIA) patients with MCA stenosis. This may improve our understanding regarding how the presence of ICAS would act on presence or progression of WMC, and may provide therapeutic targets for prevention of the symptoms subsequent to severe WMC.

## Methods

### Subjects

This was a cross-sectional study based on the Chinese Intracranial Atherosclerosis (CICAS) study (13). CICAS was a prospective, multicenter, hospital-based, cohort study carried out in 22 hospitals covering a wide geographic area throughout China, enrolling non-cardioembolic ischemic stroke or TIA patients (aged 18–80 years) admitted within 7 days of ictus (13). All patients underwent 1.5- or 3.0-T MR exams, including brain and vascular imaging sequences. Details of the inclusion and exclusion criteria, patient demographics, clinical features, and follow-up information have been described previously (13, 14). The study had been approved by Institutional Review Board at the participating hospitals. Each patient gave written informed consent.

**Figure 1:**
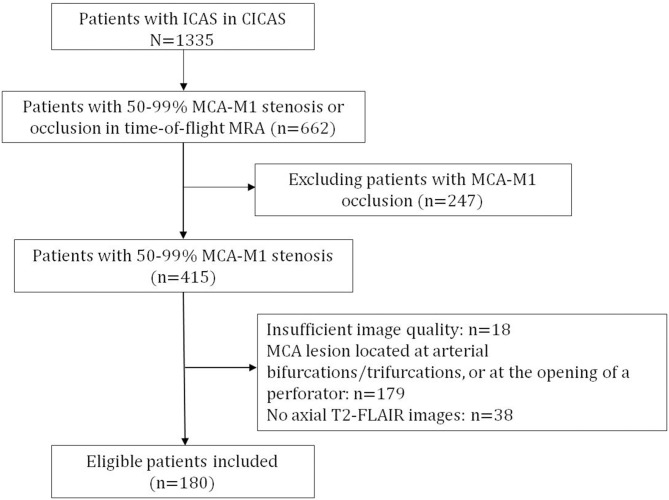
Flow chart of patient screening for the present study.

Consecutive patients recruited to the CICAS study, who had 50–99% stenosis [Warfarin-Aspirin Symptomatic Intracranial Disease (WASID) criteria (15)] of the M1 segment of unilateral or bilateral MCA (MCA-M1) in time-of-flight MRA, were screened for the current study. Those with time-of-flight MRA and T2-weighted fluid-attenuated inversion recovery (T2-FLAIR) MR images of adequate quality were enrolled and analyzed in the present study. Patients were excluded if (1) there were no T1/T2-weighted images, time-of-flight MRA or T2-FLAIR images, or the image quality was insufficient for assessment of SIR and WMC; or (2) the MCA lesion was located adjacent to an arterial bifurcation or trifurcation, or a perforator, that was not suitable for the measurement of SIR on MRA. A flow chart of patient screening and selection for the current study is provided in Figure 1. Demographics and characteristics of the index ischemic stroke or TIA were collected.

### Image Assessment

For each patient, we assessed the percentage of luminal stenosis [the WASID criteria (13, 15)] and the hemodynamic significance (by SIR) of MCA-M1 stenotic lesion in time-of-flight MRA using Phillips DICOM viewer 3.0 (Koninklijke Philips Electronics N.V.), and the severity of ipsilateral WMC by an age-related WMC (ARWMC) scale in axial T1/T2-weighted images and T2-FLAIR images using RadiAnt DICOM Viewer (Medixant). For patients with bilateral MCA-M1 stenoses, the side with a higher degree of stenosis was chosen for image assessment and subsequent analyses.

SIR was obtained in time-of-flight MRA to represent the hemodynamic significance of an MCA-M1 stenosis. The detailed methodology was described previously (10, 16). Briefly, it quantified the relative change in SIs across an ICAS lesion, adjusted by the background SI. Mean SI distal and proximal to MCA-M1 stenosis were measured on the maximum intensity projections (MIPs) showing the highest degree of stenosis of the lesion. In addition, mean background SI was calculated as the mean value of background SIs within the left and right halves of the anterior–posterior direction MIP, in areas adjacent to intracranial internal carotid arteries but free of vessel signals. SIR was then calculated as: SIR = (mean post-stenotic SI – mean background SI)/(mean pre-stenotic SI – mean background SI), with a lower SIR indicating a hemodynamically more severe lesion. The intra-rater (Pearson correlation coefficient, 0.975) and inter-rater reproducibilities (Pearson correlation coefficient, 0.847) of SIR measurement of ICAS lesions have been previously demonstrated (9, 16).

WMC was defined as ill-defined hyperintensities ≥5 mm on both T2-weighted and FLAIR imaging and isointensity on T1-weighted imaging (17, 18). The severity of WMCs was scaled in four regions in the supratentorial hemisphere ipsilateral to the index MCA-M1 stenosis, including the frontal area, the parieto-occipital area, the temporal area (scores 0–3 for each region, respectively, indicating no lesion, focal lesions, beginning confluence of lesions, and diffuse lesions), and the basal ganglia area (scores 0–3, respectively, indicating no lesion, one focal lesion, more than one focal lesion, confluent lesions) (17, 18). The ipsilesional ARWMC scale, ranging from 0 to 12, was calculated as a sum of WMC scores in the four areas, with a larger scale indicating more severe WMC. Two authors (HF and XL) independently evaluated unilateral, supratentorial ARWMC in 20 cases, for the assessment of inter-rater reproducibility of the ARWMC scale. The hemisphere for ARWMC assessment in each patient was determined before the assessment; the two investigators conducting ARWMC assessment were blinded to the data of sites and degrees of stenosis in ICAS lesions in each patient, which were centrally measured by CICAS investigators prior to this sub-study. Kappa test showed that the inter-rater reproducibility of ARWMC assessment was 0.79 for frontal lobe, 0.957 for parietal–occipital lobe, 0.773 for temporal lobe, and 1 for basal ganglia, indicating substantial agreement between the two observers.

### Statistical Analysis

Inter-rater reproducibilities of unilateral ARWMC scales of the four regions of interest were assessed by the kappa statistic, with ARWMC as an ordinal variable. Chi-square tests were conducted for the correlations of the degree of MCA-M1 stenosis (50–69% vs. 70–99% stenosis) and SIR (< or ≥ median) as dichotomized variables, with ipsilateral ARWMC scale as an ordinal variable in its quartiles. Spearman correlation coefficients and scatterplots were used to reveal linear correlations between the degree of MCA-M1 stenosis, SIR, and the ipsilateral ARWMC scale, all as continuous variables.

In addition to the degree of MCA-M1 stenosis and its hemodynamic significance by SIR, the differences in the following factors among patients with different supratentorial ARWMC scales by quartiles were also assessed in univariate analyses, including patient demographics, characteristics of the index stroke or TIA, and vascular risk factors, using chi-square tests for categorical variables and ANOVA for continuous variables. Factors with two-sided *p* < 0.1 in univariate analyses were further analyzed in a multivariate, ordinal logistic regression model, to identify factors independently associated with the ARWMC scale in quartiles. Two-sided *p* < 0.05 were considered statistically significant. All statistical analyses were performed using SAS software version 9.1 (SAS Institute Inc, Cary, NC).

## Results

### Patient Characteristics

Of the 1,335 patients with ICAS recruited to the CICAS study (13), 415 had 50–99% stenosis [WASID criteria (15)] of unilateral or bilateral MCA-M1(s) in time-of-flight MRA. After excluding 18 patients with insufficient image quality, 179 patients with stenosis located adjacent to an arterial bifurcation or trifurcation, or a perforator, and 38 patients without axial T2-FLAIR images, 180 patients were analyzed in the current study (flow chart shown in Figure 1).

Among the 180 patients, 116 were men and 64 were women, with a mean age of 63.7 ± 11.6 years. Premorbid modified Rankin scale was 0–2 in all patients. The median interval between symptom onset and admission was 2 days [interquartile range (IQR), 1–4]. The median National Institutes of Health Stroke Scale (NIHSS) upon admission was 4 (IQR 2–7). Among the patients, 126 (71.2%) had a history of hypertension, 34 (23.1%) had dyslipidemia, and 55 (31.1%) had diabetes mellitus (Table 1). No patient had a history of intracranial hemorrhage or atrial fibrillation.

**Table 1:** Univariate comparisons of clinical and imaging characteristics^*^ in patients grouped by ARWMC scales in quartiles.

**Variables**	**Overall (*n* = 180)**	**Quartile 1 (*n* = 19)**	**Quartile 2 (*n* = 63)**	**Quartile 3 (*n* = 47)**	**Quartile 4 (*n* = 51)**	***P*-value**
Age	63.7 ± 11.6	55.3 ± 10.7	61.0 ± 12.6	64.7 ± 10.8	69.4 ± 8.2	<0.001
Sex (male)	116 (64.4)	16 (84.2)	40 (63.5)	29 (61.7)	31 (60.8)	0.294
Current smoker	51 (28.3)	8 (42.1)	23 (36.5)	12 (25.5)	8 (15.7)	0.039
History of hypertension	126 (71.2)	13 (72.2)	39 (61.9)	34 (73.9)	40 (80)	0.194
History of diabetes mellitus	55 (31.1)	6 (31.6)	20 (31.8)	16 (36.4)	13 (25.5)	0.721
History of dyslipidemia	34 (23.1)	5 (35.7)	11 (21.2)	9 (22.5)	9 (22)	0.705
History of coronary heart disease	18 (11.0)	1 (6.3)	5 (8.6)	6 (14)	6 (12.8)	0.658
Prior ischemic stroke/TIA	48 (26.7)	2 (10.5)	19 (30.2)	11 (23.4)	16 (31.4)	0.289
NIHSS at admission	4 (2–7)	4 (1–6)	3 (2–7)	4 (1–8)	4 (1–7)	0.95
Premorbid mRS	0 (0–0)	0 (0–0)	0 (0–0)	0 (0–0)	0 (0–1)	0.059
Interval from onset to admission, days	2 (1–4)	3 (1–6)	2 (1–4)	2 (1–3)	1 (0–4)	0.617
Interval from onset to MRI, days	5 (2–8)	5 (1–8)	4 (2–7)	4 (2–8)	5 (3–8)	0.858
Fasting blood glucose, mmol/L	5.52 (4.7–6.9)	5.8 (4.7–7.9)	5.5 (4.8–6.7)	5.8 (4.7–8)	5.3 (4.6–6.6)	0.625
Total cholesterol, mmol/L	4.82 (4.1–5.5)	5 (4–5.4)	5 (4.2–5.6)	4.9 (4.2–5.6)	4.3 (3.9–5.1)	0.062
Triglycerides, mmol/L	1.5 (1.1–2.2)	2.4 (1.4–3.2)	1.5 (1.1–2.2)	1.4 (1.1–2.4)	1.3 (1–1.8)	0.014
High-density lipoprotein, mmol/L	1.1 (0.9–1.3)	1 (0.9–1.2)	1.1 (0.9–1.3)	1. (0.9–1.3)	1.1 (1–1.3)	0.39
Low-density lipoprotein, mmol/L	2.99 (2.38–3.67)	3.2 (2.4–3.4)	3 (2.5–3.6)	3.1 (2.6–4)	2.6 (2.1–3.4)	0.116
70–99% luminal stenosis of the index ICAS lesion	52 (28.9%)	2 (10.5%)	21 (33.3%)	19 (40.4%)	10 (19.6%)	0.031
SIR < median (0.89)	89 (49.4%)	2 (10.5%)	25 (39.7%)	18 (38.3%)	44 (86.3%)	<0.001

*TIA, transient ischemic attack; NIHSS, national institutes of health stroke scale; mRS, modified Rankin scale; MRI, magnetic resonance imaging; ICAS, intracranial atherosclerotic stenosis; SIR, signal intensity ratio*.

* *Variables are presented in means ± standard deviations, medians (interquartile ranges), or numbers (percentages)*.

### Characteristics of the Index MCA-M1 Lesions

The median interval between symptom onset and MRI examination was 2 days (IQR 1–4). Sixty-seven (37.2%) patients underwent 1.5-T MR exams and 113 (62.8%) underwent 3.0-T MR exams. Fifty-two (28.9%) and 128 (71.1%) patients had severe (70–99%) and moderate (50–69%) MCA-M1 stenosis, respectively. The mean SIR of all index MCA-M1 lesions was 0.86 ± 0.21, and the median SIR was 0.89 (IQR 0.73–0.97). The median ipsilesional, supratentorial ARWMC scale was 2 (IQR 1–2).

### Correlations of Degree of MCA-M1 Stenosis and SIR With Ipsilateral ARWMC

**Figure 2:**
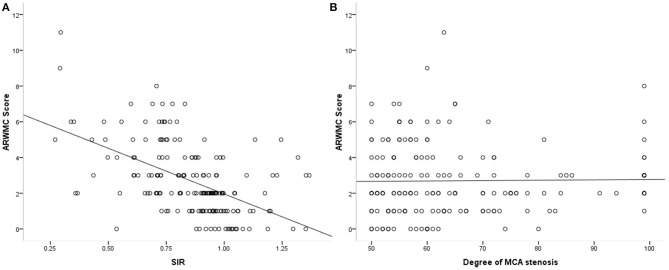
Scatterplots showing the correlations of SIR and degree of MCA stenosis (in percentage) with ipsilateral ARWMC scale.

As shown in Table 1, the proportions of cases with severe (70–99%) MCA-M1 stenosis (*p* = 0.031) and a SIR < median (*p* < 0.001) were significantly higher in those with higher ARWMC scale in univariate analyses. However, the severity of MCA luminal stenosis was not significantly, linearly related to ipsilesional ARWMC scale (Spearman correlation coefficient, 0.007; *p* = 0.93; Figure 2).

**Figure 3:**
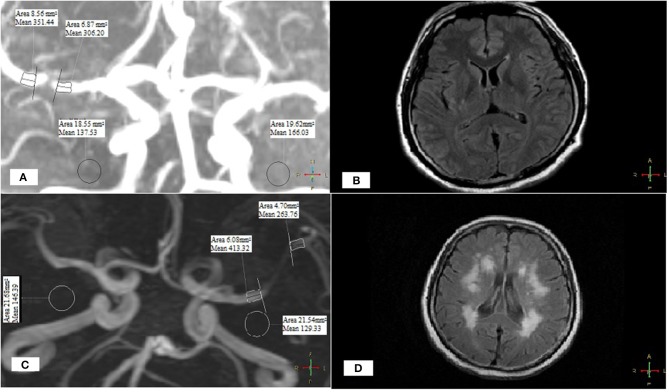
Two cases with different SIR and ARWMC scales.

The proportions of patients with SIR < median were significantly higher in those with a higher ARWMC scale (*p* < 0.001), which was 86.3% in those with an ARWMC in the highest quartile (Table 1). SIR and the ipsilateral ARWMC scale were significantly, linearly, and negatively correlated (Spearman correlation coefficient, −0.543; *p* < 0.001; Figure 2), which means that lower SIR (more hemodynamically significant) of MCA-M1 stenosis was associated with more severe ipsilesional WMC. Figure 3 shows the MRA and FLAIR images of two cases, one with a high SIR and a low ARWMC scale, and the other with a low SIR and a high ARWMC scale.

### Independent Predictors of Ipsilesional ARWMC

In addition to the severity of MCA-M1 stenosis and the SIR, other factors differed (*p* < 0.1) between patients with different ARWMC scales by quartiles in univariate analyses, including age (*p* < 0.001), current smoker (*p* = 0.039), premorbid modified Rankin Scale (*p* = 0.059), and total cholesterol (*p* = 0.062) and triglycerides (*p* = 0.014) levels (Table 1). These factors were included in multivariate, ordinal logistic regression analysis, which revealed that older age (OR = 1.037, 95% CI 1.008–1.066; *p* = 0.011) and lower SIR (OR = 0.010, 95% CI 0.002–0.058; < 0.001), but not the degree of MCA stenosis (OR = 0.725, 95% CI 0.374–1.403; *p* = 0.340), were significantly, independently associated with a higher ipsilateral ARWMC scale (Table 2).

**Table 2:** Multivariate ordinal logistic regression analysis for independent predictors of the ipsilateral ARWMC scale in quartiles.

**Variables**	**Odds ratio**	**95% confidence** **interval**	***P*-value**
Age	1.037	1.008–1.066	0.011
Current smoker	0.578	0.296–1.128	0.108
Premorbid mRS	1.316	0.738–2.347	0.351
Total cholesterol, mmol/L	0.870	0.689–1.100	0.245
Triglycerides, mmol/L	0.927	0.761–1.130	0.456
Severity of stenosis (70–99%)	0.725	0.374–1.403	0.340
SIR < median (0.89)	0.010	0.002–0.058	<0.001

*mRS, modified Rankin scale; SIR, signal intensity ratio*.

## Discussion

In the present study, we investigated whether the severity of luminal stenosis and the hemodynamic significance of MCA stenosis (as quantified by SIR in time-of-flight MRA) were associated with the severity of ipsilateral WMC (as assessed by the ARWMC scale in MRI). The results showed that SIR was significantly, linearly, and negatively correlated with ipsilateral, supratentorial ARWMC scale, and multivariate analyses further corroborated the association of lower SIR with higher ARWMC scale, independent of other confounding factors. The findings indicated that patients with hemodynamically more severe MCA stenosis were more likely to have more severe WMC in the ipsilateral hemisphere. However, the degree of luminal narrowing in MCA stenosis was not significant associated with the severity of ipsilateral WMC in this study. Of note, older age, as an established risk factor of WMC, was also found as an independent predictor of higher ARWMC scale in the current study.

For patients with ICAS, SIR could be easily obtained with the wide application of time-of-flight MRA in clinical practice. Based on the contrast mechanism (flow-related enhancement) of time-of-flight MRA, a lower SIR of an ICAS lesion could, to some extent, reflect downstream hemodynamic impairment caused by the stenotic lesion, which has been correlated with prolonged cerebral perfusion (12), larger acute infarct volume (10), and higher risk of recurrent stroke (11), in patients with a recent ischemic stroke or TIA in previous studies. With previously demonstrated intra- and inter-rater reproducibilities (16), the index SIR could be considered as a simple and effective marker to gauge the hemodynamic significance of ICAS, to be used in relevant research areas. On the other hand, for inter-rater reproducibility of ARWMC assessment in the current study, the kappa test result was 0.79 for frontal lobe, 0.957 for parietal–occipital lobe, 0.773 for temporal lobe, and 1 for basal ganglia, indicating substantial agreement between two observers, which had been demonstrated in previous studies (18). Therefore, this ARWMC assessment scale is operational in clinical practice for assessment of severity of WMC (18).

The roles of extracranial and intracranial large artery atherosclerosis in the development and progression of WMC are uncertain. As mentioned earlier, previous studies showed conflicting results concerning the correlations of presence/extent of WMC with different stages of carotid atherosclerosis (increased intima-media thickness, non-stenotic and stenotic carotid plaques) and intracranial atherosclerosis (mostly intracranial stenosis) (5–8). Some of the studies reported significant correlations while others not. The current study indicated that it might be the hemodynamic impact of ICAS lesions and the possibly subsequent hypoperfusion, but not the degree of luminal stenosis, that governs the severity of ipsilateral WMC. Such inferences are supported by significant correlations between larger white matter lesion volumes and reduced total cerebral blood flow in previous large cohort studies (19–21). Compared with the cortex, the white matter might be more vulnerable to hemodynamic insufficiency, which is supplied by single source subependymal arteries with scarce anastomoses (22–24). This may partly explain the conflicting results in previous studies: the diverse hemodynamic severities of extra- and intra-cranial atherosclerotic lesions in those studies might have interfered with the analyses of their correlations with WMC.

The current findings in the important role of hemodynamic impact of ICAS in determining the severity of WMC may have clinical implications in the management of ICAS patients. As mentioned above, WMC have been related to increased risks of cognitive impairment, dementia, and stroke (1–4). Thus, in this regard, patients with ICAS may benefit from hemodynamic restoring treatment methods, such as angioplasty/stenting, and other treatment methods such as external counter pulsation that can enhance cerebral perfusion (25, 26). However, these inferences need further investigations in future prospective studies.

This study had several limitations. First, patients who underwent 1.5- and 3.0-T MR exams were both included in the present study. The relatively inferior quality of 1.5-T MR images might, to some extent, interfere with the evaluation of SIR and ARWMC, and the severity of MCA luminal stenosis may be overestimated with time-of-flight MRA (27). Second, the influence of isolated or concurrent extracranial stenosis was not considered in the present study, which may be a potential confounder in the correlations between ICAS and WMC. Third, treatment strategies such as medications of the patients receiving may interfere with the associations between SIR and ARWMC, which were not adjusted in the multivariate ordinal logistic regression analyses. Last but not least, the flow-dependent artery visualization mechanism of time-of-flight MRA prohibits accurate calculation of SIR when an ICAS lesion is located adjacent to arterial bifurcations, trifurcations, or perforators, in which case the SIs may alter with or without the presence of ICAS. A considerable number of patients were excluded from the current study due to this reason, which may arouse selection bias.

In conclusion, older age and lower SIR, but not the percentage of luminal stenosis, were independent predictors of higher ipsilateral ARWMC in stroke or TIA patients with MCA-M1 stenosis. In other words, in patients with atherosclerotic MCA stenosis, the hemodynamic impact of the stenotic lesion, rather than the severity of luminal narrowing, may partly determine the severity of ipsilateral WMC. However, it is unclear how the hemodynamic significance of ICAS affects the development and progression of white matter changes based on this cross-sectional study. Further longitudinal studies with sequential imaging exams are warranted.

## Data Availability Statement

All datasets generated for this study are included in the article/supplementary material.

## Ethics Statement

The studies involving human participants were reviewed and approved by Ethics Committee of the Tiantan Hospital. The patients/participants provided their written informed consent to participate in this study.

## Author Contributions

All authors participated in the interpretation of study results and in the drafting, critical revision, and approval of the final version of the article. HF and XL was responsible for study design, data analyses, and drafting and revising the article. HF was responsible for acquisition of data, data analyses, and drafting the article. YPu, XZo, BS, YS, TL, CW, XZh, YiW, and YoW were involved in revising the article for important intellectual content. YPa conducted the statistical analysis. LL and YX was responsible for study concept or design, technical, material support, administrative, and supervision.

### Conflict of Interest

The authors declare that the research was conducted in the absence of any commercial or financial relationships that could be construed as a potential conflict of interest.
